# Activated protein C consumption and coagulation parameters in severe sepsis and septic shock

**DOI:** 10.1186/cc13416

**Published:** 2014-03-17

**Authors:** M De La Torre-Prados, A Garcia-de la Torre, C Trujillano-Fernandez, J Perez-Vacas, A Puerto-Morlan, E Camara-Sola

**Affiliations:** 1 Hospital Virgen de la Victoria, Málaga, Spain; 2 University Hospital Puerto Real, Cadiz, Spain

## Introduction

Activated protein C (APC) deficiency is prevalent in severe sepsis and septic shock patients. The aim of the study was to relate the anticoagulation activity evaluated by APC with other coagulation parameters adjusted to 28-day mortality.

## Methods

A cohort study of 150 critically ill adults. Age, sex, sources of infection and coagulation markers within 24 hours from severe sepsis or septic shock onset, defined according to Surviving Sepsis Campaign (SSC) criteria, were studied. We analyzed APC activity using a hemostasis laboratory analyzer (BCS^® ^XP; Siemens). A descriptive and comparative statistical analysis was performed using SPSS version 15.0 (SPSS Inc., Chicago, IL, USA).

## Results

**Figure 1:**
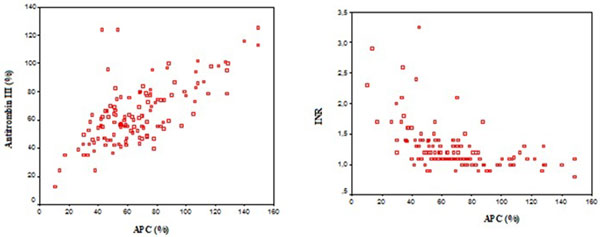
**Relationship between activated protein C and antithrombin III and INR**.

We analyzed 150 consecutive episodes of severe sepsis (16%) or septic shock (84%) admitted to the UCI. The median age of the study sample was 64 (interquartile range (IQR): 22.3 years; male: 60%). The main sources of infection were: respiratory tract 38%, intra-abdomen 45%, and 70.7% had medical pathology. The 28-day mortality was 22.7%. Nonsurvivors had a significantly higher consumption of APC than survivors, 56% (IQR: 38.5) versus 68.6 (IQR: 41.4); *P *= 0.023. The profile of lower levels of APC was a surgery patient with septic shock, neurological focus or catheter-related infection and Gram-negative pathogens from blood cultures. Spearman's showed relationship with antithrombin III, *r *= 0.674 (*P *< 0.001) and International Normalized Ratio (INR), *r *= -0.611 (*P *> 0.001). See Figure 1.

## Conclusion

Low levels of PC are associated with poor outcome and severity in severe sepsis, and it is well correlated with antithrombin III and INR.

